# Impaired Interference Control in Individuals With Internet Addiction: Evidence From Event‐Related Potentials and Brain Oscillations

**DOI:** 10.1111/adb.70062

**Published:** 2025-07-24

**Authors:** Farzad Rostami, Ali Esteki, Sepideh Khoniveh, Rana Ghamari, Atiye Sarabi‐Jamab

**Affiliations:** ^1^ School of Cognitive Sciences Institute for Research in Fundamental Sciences (IPM) Tehran Iran; ^2^ CNRS UMR5229–Institut des Sciences Cognitives Marc Jeannerod Bron France; ^3^ Department of Biomedical Engineering and Medical Physics, Faculty of Medicine Shahid Beheshti University of Medical Sciences Tehran Iran; ^4^ University Medical Center Hamburg‐Eppendorf Institute for Systematicneuroscience Hamburg Germany; ^5^ Department of Cellular and Molecular Biology, Faculty of Biological Sciences Kharazmi University Tehran Iran; ^6^ Faculty of Governance University of Tehran Tehran Iran

**Keywords:** conflict, EEG, ERP, IAD, interference control, time–frequency analysis

## Abstract

Individuals with Internet addiction disorder (IAD) exhibit deficits in cognitive control, particularly in interference control; however, the behavioural and neural mechanisms underlying these impairments remain unclear. In this study, classic and modified Stroop tasks were administered to individuals with IAD and healthy control (HC) participants, whereas electroencephalography (EEG) was recorded. We hypothesized that individuals with IAD would demonstrate impaired interference control, as evidenced by longer reaction times (RTs) on incongruent trials and that these behavioural deficits would be accompanied by reduced ERP activity in both early and late medial frontal negativity (MFN) components, as well as diminished conflict slow potential (SP). Additionally, our event‐related spectral perturbation (ERSP) analysis was designed to examine oscillatory dynamics, including a reduced Stroop effect in theta power along with compensatory increases in beta2 and gamma band activity. The results revealed that individuals with IAD exhibited prolonged RTs, with this difference becoming more pronounced under increased cognitive demands. Furthermore, ERP responses and ERSP patterns across frequency bands were distinct in the IAD group, pointing to deficits in conflict detection and resolution, as well as compensatory neural mechanisms. These findings suggest that cognitive slowing in individuals with IAD is exacerbated under conditions requiring greater interference control, contributing to the executive dysfunction.

## Graphical abstract

Individuals with Internet Addiction Disorder (IAD) exhibit pronounced deficits in interference control, shown by increased reaction times under cognitively demanding tasks. EEG analyses reveal impaired neural activity, characterised by reduced early and late medial frontal negativity (MFN) and diminished slow potential (SP), signalling deficits in conflict detection and resolution. Furthermore, altered oscillatory dynamics—decreased theta power with compensatory increases in beta2 and gamma activity—highlight neural processing impairments that exacerbate cognitive slowing under conditions requiring enhanced executive control.

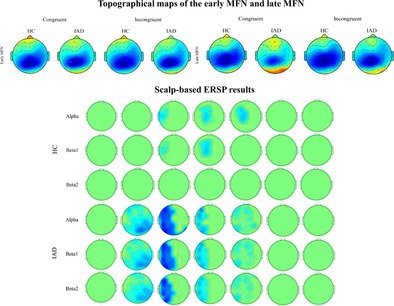

## 1 Introduction

In the digital era, ‘Internet addiction’ (IA) has emerged as a new behavioural addiction, with widespread Internet access and events like the COVID‐19 pandemic increasing dependency on cyberspace [1]. Individuals with Internet addiction disorder (IAD) exhibit compulsive internet use that negatively impacts physical and mental health, leading to a significant decline in psychological and social functioning [2]. Like other behavioural addictions—including gaming and social networking—and substance use disorders [3, 4, 5], IAD is linked to impairments in cognitive control [6].

Previous studies have found that individuals with IAD often experience deficits in cognitive functions, including error processing [5], attention [7], memory [8], executive function [9, 10], perception [11], inhibitory control [9], interference resolution [7] and decision making [12]. Deficient interference control, a subset of inhibitory control, is particularly pronounced in IAD and contributes to difficulties in resolving conflicts between competing stimuli or responses [13]. These deficits are evident in cognitive tasks such as the Stroop task, where participants with IAD exhibit increased reaction times (RTs) and error rates in interference conditions, reflecting diminished cognitive flexibility and attentional regulation [9, 10]. However, although previous studies generally report increased RTs in IAD individuals, findings regarding error rates have been more inconsistent. Some studies suggest that IAD individuals commit more errors, whereas others indicate that they engage in compensatory mechanisms by slowing down their responses to maintain accuracy [12, 13]. Such behavioural impairments mirror those observed in individuals with substance use disorders and may underlie the compulsive and maladaptive internet use characteristic of IAD [14].

Inhibitory control, the ability to override strong internal or external predispositions and control attention, behaviour, thoughts or emotions, is often less efficient in individuals with IAD compared to the healthy control (HC) group [9, 10, 12, 13]. Deficiencies in interference control and inhibitory impairment may lead to conflicts between stimulus and response selection, involving the simultaneous activation of incorrect and correct response selections or two competing stimulus types [15]. This conflict is central to the conflict monitoring theory, which posits that the brain continuously monitors for such conflicts and adjusts behaviour accordingly. These deficits are also observed in individuals with psychiatric disorders such as depression, anxiety or obsessive–compulsive disorder and are often targeted in pharmacological or behavioural treatments [14, 16].

Participants with IAD show reduced inhibitory control compared to the HC group, particularly in interference control, which involves resolving conflicts between competing stimuli or responses [17]. Neuroimaging techniques such as functional magnetic resonance imaging (fMRI) and electroencephalography (EEG) have been widely used to investigate the neural mechanisms underlying these deficits. Specific event‐related potential (ERP) studies have demonstrated that error‐related negativity (ERN) reflects response conflict [18], and the early and late medial frontal negativity (MFN) components serve as markers of conflict monitoring and executive control [19, 20]. These deficits have been linked to neural activity in the anterior cingulate cortex (ACC) and the medial frontal cortex, highlighting their role in ACC‐mediated conflict monitoring [21].

Stimulus‐locked ERP studies have shown that the slow potential (SP) is sustained positively over the parietal lobe and reflects cognitive control adjustment (conflict adjustment). The SP over the lateral frontal area and the posterior cortices exhibits positive activity that follows the late MFN, indicating increasing recruitment of cognitive control resources and compensatory processes to avoid error commission [22].

Individuals with IAD exhibit a reduced ability to inhibit impulsive behaviours, which may be linked to disruptions in activity and functional connectivity within the prefrontal cortex (PFC). These alterations in prefrontal functioning have been associated with impairments in self‐regulation and cognitive control. This impairment is reflected in altered amplitudes in the N2 and P3 components during conflict detection, indicating a greater demand for attentional resources and a reduced ability to monitor conflicts [10, 23]. The N2 component is associated with early conflict detection and response inhibition, whereas the P3 reflects attentional allocation and conflict resolution, with alterations in both indicating deficits in detecting and resolving cognitive conflict [24].

Studies on brain oscillations and addiction have revealed the involvement of delta and theta bands in signal detection, decision‐making and inhibitory processes [25, 26]. Theta power, in particular, has been associated with successful conflict resolution [27], but individuals with substance use disorders demonstrate significantly reduced delta and theta power during no‐go trials, indicating impaired inhibition mechanisms and information processing [28]. Alterations in the frontal alpha band among individuals with IAD may indicate a deficit in inhibitory control [29]. The alpha band is thought to play a crucial role in top‐down inhibitory control by suppressing task‐irrelevant information and regulating attentional focus [30]. Reduced frontal alpha power has been associated with impaired suppression of distractions and diminished executive control, further supporting its relevance in cognitive regulation deficits observed in IAD [31]. Additionally, reductions in beta band power and gamma band strength have been reported in individuals with IAD, further supporting the view that IAD involves widespread neural alterations [32].

Our study focused on investigating the impact of IAD on cognitive control processes, specifically in the context of conflict monitoring. A key limitation of prior research is that traditional interference tasks, such as the classic Stroop task, may not fully capture the extent of interference control impairments in IAD due to participants' ability to adapt to task conditions over time. This adaptation reduces the sensitivity of standard Stroop tasks in detecting subtle impairments in cognitive control. To address this issue, we employed a modified version of the Stroop task that systematically enhances cognitive interference by reducing short‐term priming effects. This modification ensured that the ink colour dimension remained unpredictable based on previous trials, thereby increasing the demands on interference control mechanisms. The rationale behind this modification was to minimize the potential for adaptation effects that might otherwise obscure group differences in cognitive control performance [33]. By disrupting sequential trial facilitation, our task design created a more demanding interference control context, allowing us to better assess inhibitory control deficits in individuals with IAD. We assessed ERPs and event‐related spectral perturbations (ERSPs) to evaluate inhibitory control deficits—with a particular emphasis on interference control—in individuals with IAD. Behaviourally, we hypothesized that participants with IAD would exhibit longer RTs on incongruent trials relative to the HC group, reflecting increased cognitive effort required to resolve interference. However, we did not necessarily expect higher error rates, as prolonged response times may indicate a compensatory mechanism wherein IAD individuals delay their responses to maintain accuracy.

Neurally, we hypothesized that the IAD group would exhibit reduced ERP activity in both the early and late MFN components—reflecting diminished conflict detection and resolution capabilities—and decreased activity in the conflict SP, indicative of impaired compensatory cognitive control adjustment processes. The ERSP analysis enabled us to examine changes in oscillatory power across frequencies and time intervals, thereby illuminating the dynamic neural mechanisms underlying interference control [34].

Overall, our study hypothesized that individuals with IAD would demonstrate impairments in cognitive control, resulting in increased RTs in both the traditional and modified Stroop tasks. By employing the modified Stroop task, we aimed to reveal more nuanced differences in cognitive control between the IAD and HC groups, particularly in how interference resolution is impacted by task demands. Additionally, we expected a general reduction in neural resource activation in the IAD group and a decrease in left‐lateralized prefrontal asymmetry associated with interference control and Stroop performance in the HC group. Our study aimed to contribute to a deeper understanding of the neural mechanisms underlying interference control deficits in IAD and the compensatory strategies that may mask these impairments in standard behavioural measures.

## 2 Materials and Methods

### 2.1 Participants

Participants were recruited from universities in Tehran, Iran, through an online advertisement. Initially, 2000 individuals completed the Internet Addiction Test (IAT), developed by Young [35], questionnaire (see Section 2.2 for threshold criteria). Those scoring above 70 were invited for further screening by a trained psychiatrist to exclude participants with depression, anxiety or other emotional disorders. After screening, 21 individuals with IAD were selected and matched with 21 HC participants scoring below 30 on the IAT and having no history of anxiety or emotional disorders. The IAD and HC groups were age‐matched during participant selection to ensure comparability. All participants were right‐handed (to control for potential hemispheric differences in EEG activity), had normal or corrected‐to‐normal vision and reported no history of neurological disorders or substance use disorder. One female participant from the HC group was excluded due to technical problems with EEG recording, resulting in a final sample of 21 participants with IAD and 20 in the HC group.

### 2.2 Measures

Several standardized questionnaires were administered to assess IA, anxiety, depression and impulsivity. The IAT is a 20‐item questionnaire that measures the severity of IA on a 5‐point Likert scale (Cronbach's α = 0.90–0.93; [36]). Higher scores indicate greater levels of IA; individuals scoring above 70 were classified as having IAD, whereas those scoring below 30 were classified as belonging to the HC group. This stricter cut‐off was chosen to select participants from the upper and lower quartiles of the IAT distribution, thereby ensuring robust group differentiation and minimizing classification ambiguity [36] To assess anxiety levels, the Beck Anxiety Inventory [37], a 21‐item self‐report inventory, was used. Participants scoring below 15 were included to ensure the exclusion of those with significant anxiety. Depressive symptoms were evaluated using the Beck Depression Inventory II [38], a 21‐item self‐report questionnaire. Only participants scoring below 19 were selected to ensure they did not have significant depressive symptoms. Additionally, the Barratt Impulsiveness Scale (BIS‐11) [39], a 30‐item questionnaire assessing impulsiveness, was administered to evaluate impulsivity levels, with scores included in the demographic analysis (see Table 1).

**TABLE 1:** Demographic parameters (means and SD).

Participant groups	IAD	HC
Tasks		
Age	23.50 (4.78)	24.60 (4.21)
IAT	75.36 (5.18)	28.05 (5.65)
Impulsivity	43.95 (7.11)	37.05 (5.20)

### 2.3 Procedure

Participants performed two cognitive tasks: a **classic Stroop task (Task1)** and a **modified Stroop task (Task2)**, both programmed in MATLAB using Psychophysics Toolbox Version 3. The classic Stroop task presented 288 trials (divided into four runs), in which colour words (e.g., ‘RED’, ‘GREEN’, ‘BLUE’, ‘YELLOW’) were displayed in either congruent or incongruent ink colours. In the **modified Stroop task**, 144 trials (two runs) were presented such that the same ink colour never appeared consecutively, increasing interference demands by minimizing short‐term priming.

**FIGURE 1:**
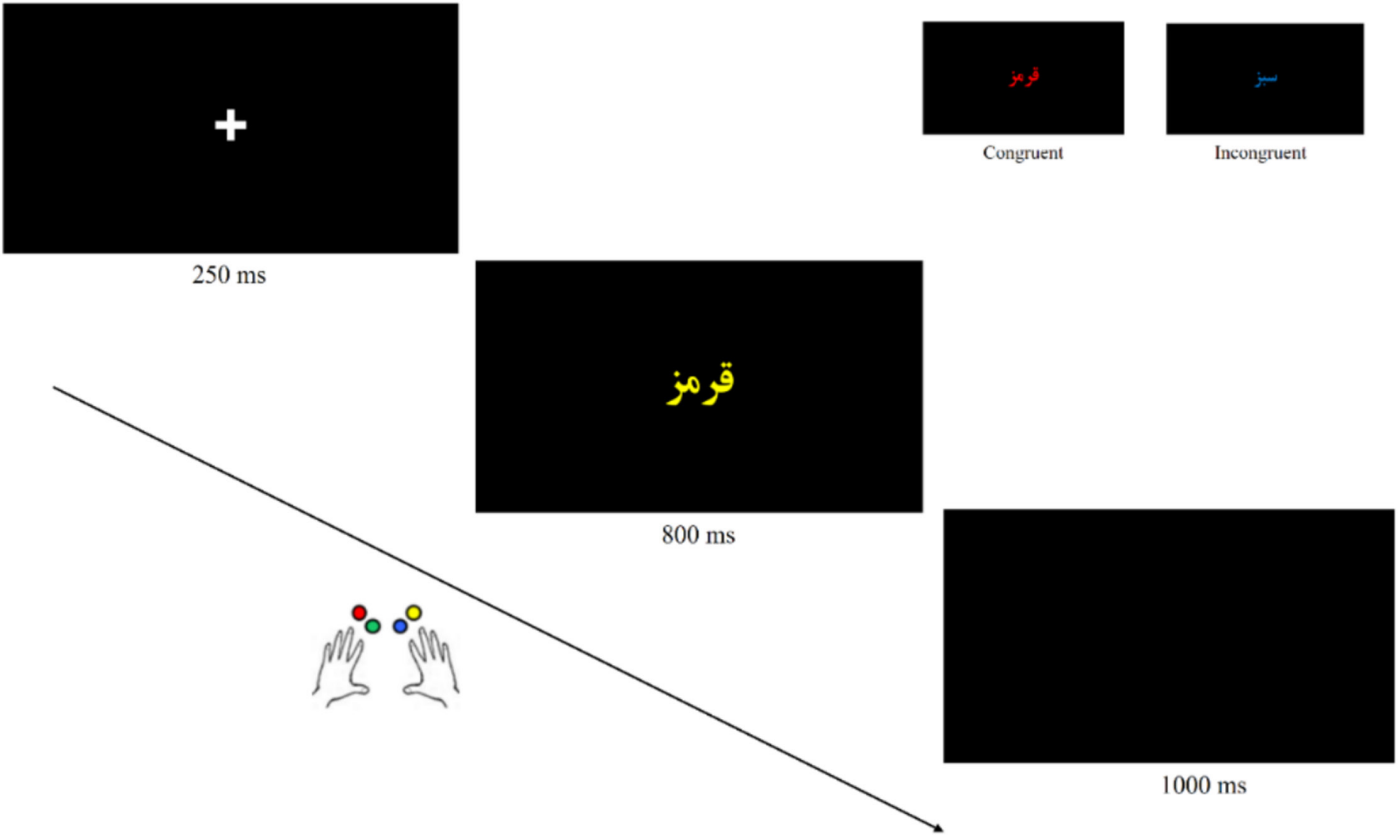
Depiction of the Stroop task process, presenting two distinct trial types on a black screen: **congruent trials**, where the word colour matches the word itself (e.g., the term ‘GREEN’ displayed in Farsi with green font colour), and **incongruent trials**, where the word colour does not correspond to the word (e.g., the term ‘GREEN’ presented in Farsi with yellow font colour).

All trials followed a similar timeline: a fixation cross appeared for 250 ms, followed by an 800 ms colour word display. Participants had up to 800 ms to respond by pressing one of four designated keys corresponding to the ink colour. A black screen was shown for 1000 ms before the next trial (Figure 1). Each participant received 16 practice trials before beginning Task1, then completed Task2. They were instructed to respond as quickly and accurately as possible.

### 2.4 EEG Recording and Preprocessing

EEG data were recorded using a 64‐channel g.HIamp amplifier (g.tec) at a 1200 Hz sampling rate, with electrodes positioned via the 10–10 system. The right mastoid served as the online reference, and the ground electrode was placed at Fpz. Electrode impedances were maintained below 10 kΩ. EEG data were preprocessed using EEGLAB v.2021.0, including band‐pass filtering (0.5–40 Hz), independent component analysis (ICA) for artefact removal and interpolation of noisy channels. Data were downsampled to 256 Hz. Epochs spanning −200–1500 ms relative to stimulus onset were extracted, baseline‐corrected using the pre‐stimulus interval, and trials exceeding ±100 μV were excluded. On average, 122 trials per condition in Task 1 and 61 trials per condition in Task 2 were retained, ensuring high data quality.

### 2.5 ERP Analysis

ERPs were computed by averaging artefact‐free epochs for each participant, condition (congruent and incongruent) and task (Task 1 and Task 2). Three components linked to conflict processing were analysed: early MFN (250–400 ms), late MFN (400–550 ms) and SP (550–900 ms). The early and late MFN components were analysed at frontal/fronto‐central electrodes (F1, F2, Fz, FC1, FC2, FCz and Cz), whereas the SP component was analysed at centro‐parietal/parietal electrodes (CP3, CPz, CP4, P3, Pz and P4).

### 2.6 ERSP Analysis

Time–frequency analysis was conducted using Morlet wavelet transformation (4–40 Hz, 1 Hz resolution). The number of wavelet cycles increased linearly (2 cycles at 4 Hz to 20 cycles at 40 Hz). Baseline correction was applied using the pre‐stimulus interval. Analyses focused on cognitive control‐relevant bands: theta (4–7 Hz), alpha (8–12 Hz), beta1 (13–19 Hz), beta2 (20–30 Hz) and gamma (31–40 Hz). ERSP differences between the IAD and HC groups were identified using cluster‐based random permutation tests [40].

### 2.7 Statistical Analysis

Statistical analyses were performed using R (v.3.6.1) in RStudio (v.1.2.5001), with *p* < 0.05 set for significance and effect sizes reported as partial eta squared ($\eta_{p}^{2}$). Behavioural data (reaction time, individual standard deviation [ISD] and error rates) were analysed via three‐way mixed‐design ANOVAs (group, congruity and task). Post hoc analyses with Bonferroni correction were performed to explore significant main effects and interactions. ERP mean amplitudes were subjected to similar ANOVAs. ERSP data were analysed via cluster‐based permutation tests, controlling for multiple comparisons.

Pearson correlations examined relationships between IAT scores and the Attentional Impulsivity subscale of the BIS‐11, investigating whether higher levels of IA were associated with increased attentional impulsivity.

### 2.8 Ethics

The study adhered to the Declaration of Helsinki, with informed consent obtained from all participants. Approval was granted by the ethics committee of Shahid Beheshti University of Medical Sciences, Tehran, Iran.

## 3 Results

### 3.1 Demographic Characteristics

An overview of participants' demographic details and IAT scores is presented in Table 1. A significant positive correlation was observed between IAT and impulsivity scores (*r* = 0.42, *p* < 0.001). A Mann–Whitney *U* test confirmed no significant difference in age between the groups (*p* > 0.05), ensuring comparability. Additionally, a Mann–Whitney *U* test revealed a significant difference in attentional impulsivity between the groups (*p* < 0.05), with individuals in the IAD group showing higher impulsivity levels than those in the HC group.

### 3.2 Mean RT

**FIGURE 2:**
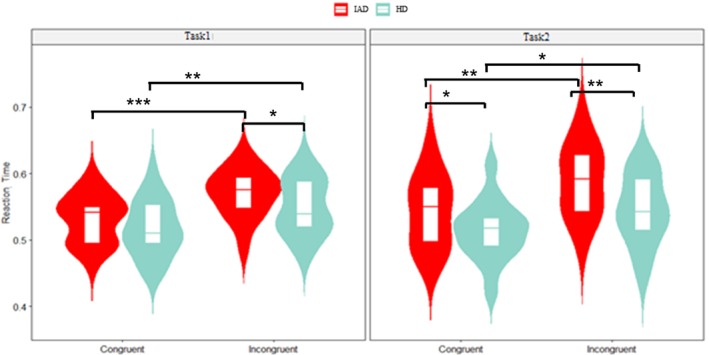
The combined violin plot and boxplot display the mean RT comparison across congruent and incongruent trials, as well as Task1 and Task2 conditions between the IAD and HC groups (*p < 0.05, ***p < 0.01, ***p < 0.001).

A three‐way ANOVA revealed a significant main effect of group, with the IAD group showing longer RTs (580 ms (SD = 110)) than the HC group (520 ms (SD = 91)) (*p* < 0.001, $\eta_{p}^{2}$ *=* 0.088). A significant main effect of congruity was found, with longer RTs in incongruent (590 ms (SD = 93)) than congruent trails (525 ms (SD = 78)) (*p* < 0.001, $\eta_{p}^{2}$ = 0.227). Figure 2 illustrates these RT differences.

### 3.3 ISD

**FIGURE 3:**
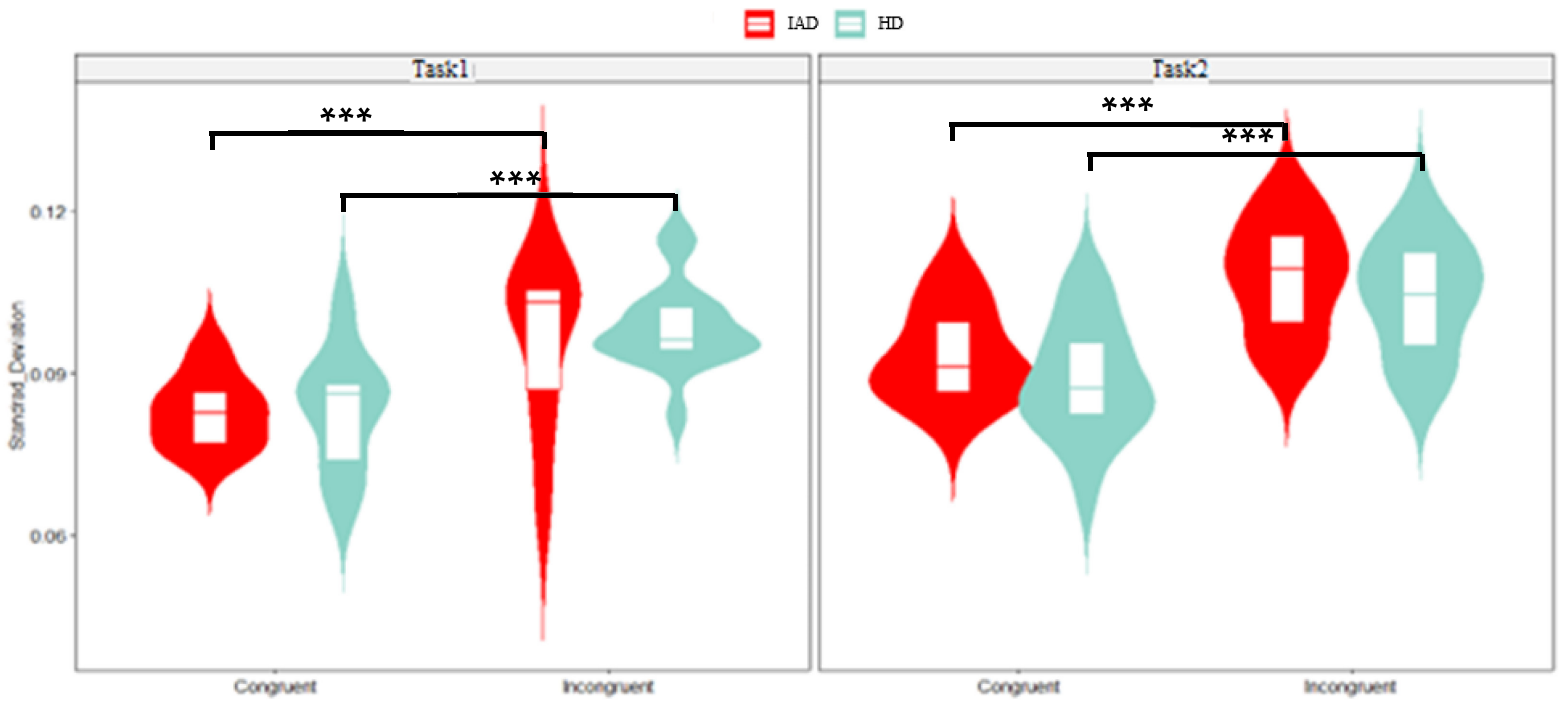
The combined violin plot and boxplot display the ISD across congruent and incongruent trials, as well as Task1 and Task2 conditions, between IAD and HC groups (*p < 0.05, **p < 0.01, ***p < 0.00).

A three‐way ANOVA revealed a significant main effect of task (*p* < 0.001, $\eta_{p}^{2}$ = 0.089), with lower ISD (i.e., reduced speed variability) in Task1 compared toTask2. A significant main effect of congruity (*p* < 0.001, $\eta_{p}^{2}$ = 0.310) indicated greater variability in incongruent than congruent trials. However, the interaction between group, congruity and task was not significant. Figure 3 presents ISD distributions.

### 3.4 Error Rate

A three‐way ANOVA showed that error rates were influenced by task difficulty and stimulus congruity. More errors occurred in incongruent than congruent trials (*p* < 0.001, $\eta_{p}^{2}$ = 0.202) and in Task2 compared to Task1 (*p* = 0.041, $\eta_{p}^{2}$ = 0.045). However, group differences were not significant (*p* = 0.214, $\eta_{p}^{2}$ = 0.032), and no interaction was found, indicating that these effects were consistent across groups.

### 3.5 ERP Data

**FIGURE 4:**
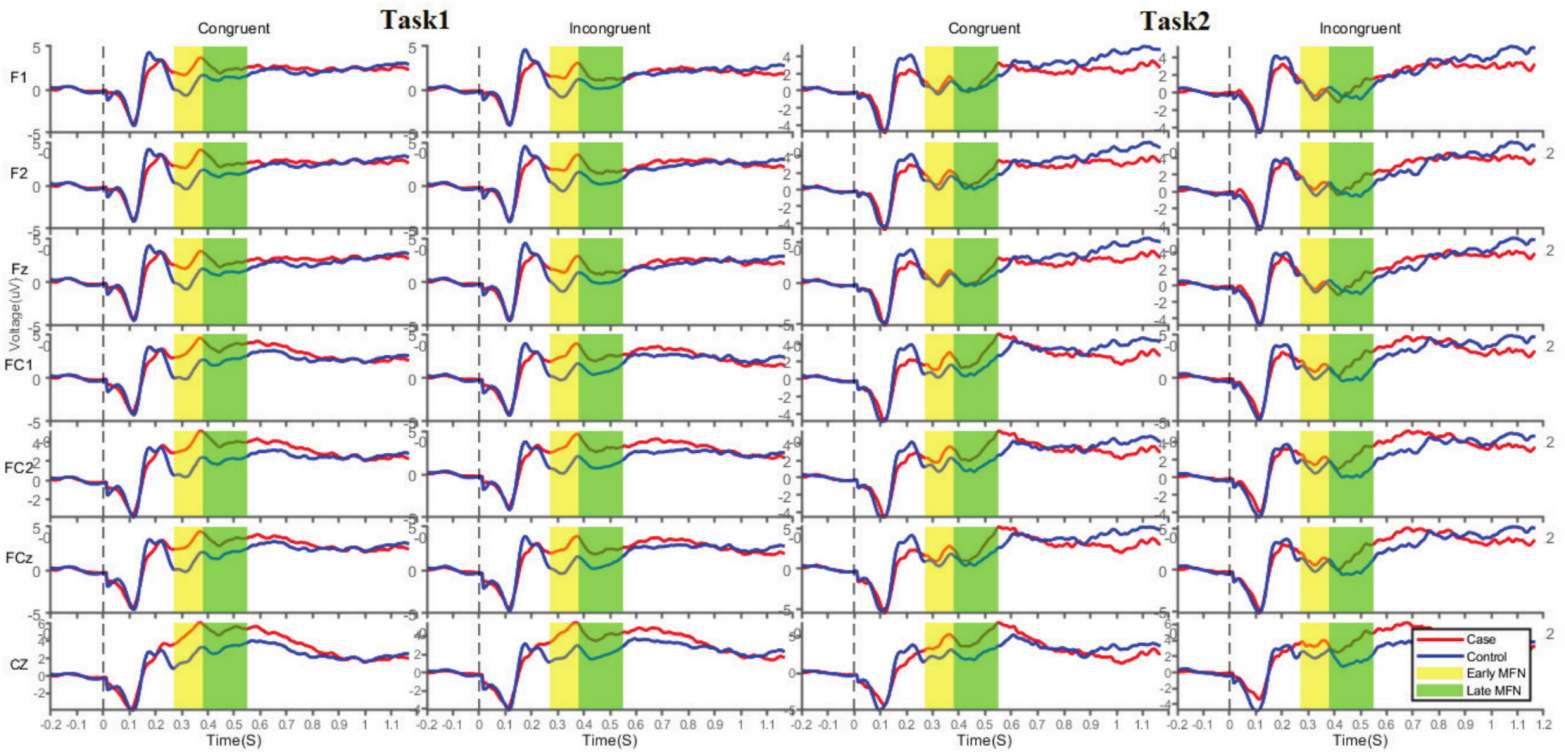
The grand average ERPs over the seven frontal electrodes (F1, F2, Fz, FC1, FC2, FCz and Cz) for the IAD group and HC group during congruent and incongruent trials, with yellow and green highlights indicating early MFN and late MFN, respectively.

**FIGURE 5:**
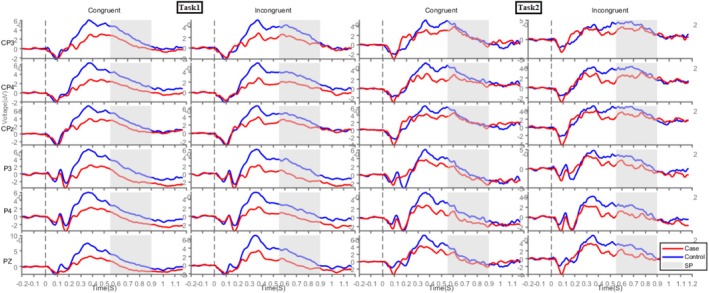
The grand average ERPs over centro‐parietal and parietal electrodes (CP3, CP4, CPz, P3, P4 and Pz) for the IAD and HC groups during congruent and incongruent trials in Task1 and Task2, with a grey highlight indicating SP.

**FIGURE 6:**
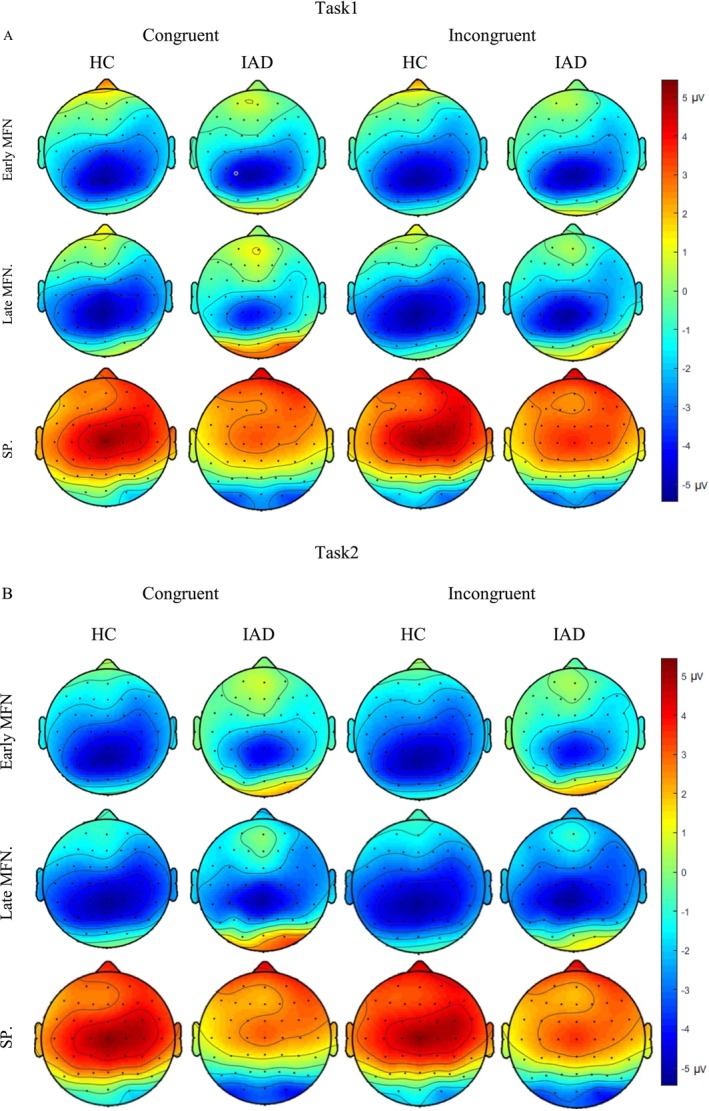
Topographical maps of the early MFN (250–400 ms), late MFN (400–550 ms) and SP (550–900 ms) in Task1 (A) and Task2 (B).

Grand‐averaged ERPs were analysed for early MFN (250–400 ms), late MFN (400–550 ms) and SP (550–900 ms) components [41, 42]. The grand‐averaged waveforms for the MFN and SP components are presented in Figures 4 and 5, respectively. The early and late MFN components were measured over frontal, fronto‐central and central electrodes, whereas the SP component was analysed over centro‐parietal and parietal electrodes [43]. Figure 6 presents topographical maps of these components across both tasks.

### 3.6 Early MFN

**FIGURE 7:**
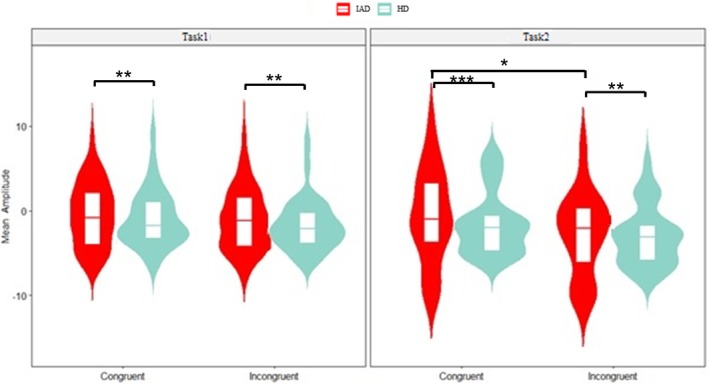
Violin plot with boxplot displaying the mean amplitude of the early MFN for congruent and incongruent trials in Task1 and Task2, comparing the IAD and HC groups (*p < 0.05, **p < 0.01, ***p < 0.001), based on data from electrodes F1, F2, Fz, FC1, FC2, FCz and Cz.

The three‐way ANOVA revealed significant main effects for group (*p* < 0.001, $\eta_{p}^{2}$ = 0.062) and task (*p* = 0.037, $\eta_{p}^{2}$ = 0.004) in the early MFN component. The IAD group showed lower negative mean amplitudes than the HC group, and Task1 exhibited a lower negative mean amplitude than Task2. Additionally, a significant congruity effect was observed (*p* = 0.046, $\eta_{p}^{2}$ = 0.004) (Figure 7).

### 3.7 Late MFN

**FIGURE 8:**
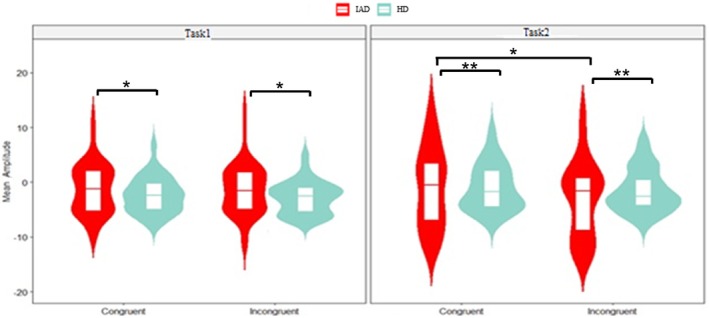
Violin plot with boxplot displaying the late MFN mean amplitude for congruent and incongruent trials in Task1 and Task2, comparing the IAD group and HC group (*p < 0.05, **p < 0.01, ***p < 0.001), based on data from electrodes F1, F2, Fz, FC1, FC2, FCz and Cz.

The three‐way ANOVA revealed a significant group effect (*p* < 0.001, $\eta_{p}^{2}$ = 0.027), with the IAD group showing lower negativity than the HC group. A significant main effect of task (*p* = 0.009, $\eta_{p}^{2}$ = 0.007), indicated lower negativity in Task1 than Task2. A significant congruity effect was also observed (*p* = 0.006, $\eta_{p}^{2}$ = 0.008) (Figure 8).

### 3.8 SP

**FIGURE 9:**
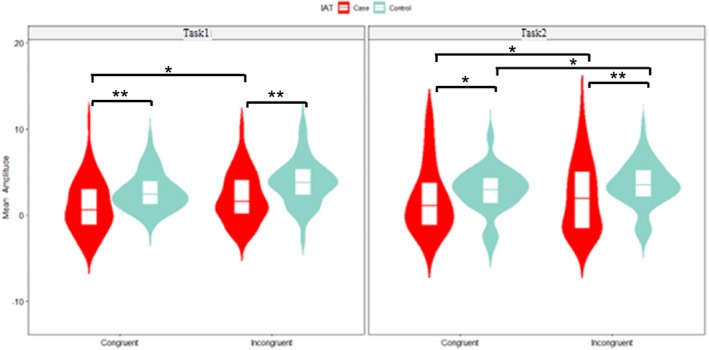
Violin plot displaying the mean amplitude of the SP component in congruent and incongruent trials in Task1 and Task2, comparing the IAD group and HC group (*p < 0.05, **p < 0.01, ***p < 0.001), based on data from electrodes CP3, CP4, CPz, P3, P4 and Pz.

The three‐way ANOVA revealed a significant group effect (*p* < 0.001, $\eta_{p}^{2}$ = 0.050), with the IAD group showing lower mean SP amplitudes than the HC group. A significant congruity effect (*p* = 0.006*,* $\eta_{p}^{2}$ = 0.009) indicated higher amplitudes in incongruent than congruent trials. A significant task effect (*p* = 0.015, $\eta_{p}^{2}$ = 0.0081) showed lower negativity in Task1 than Task2 (Figure 9).

### 3.9 Scalp‐Based Time–Frequency

**FIGURE 10:**
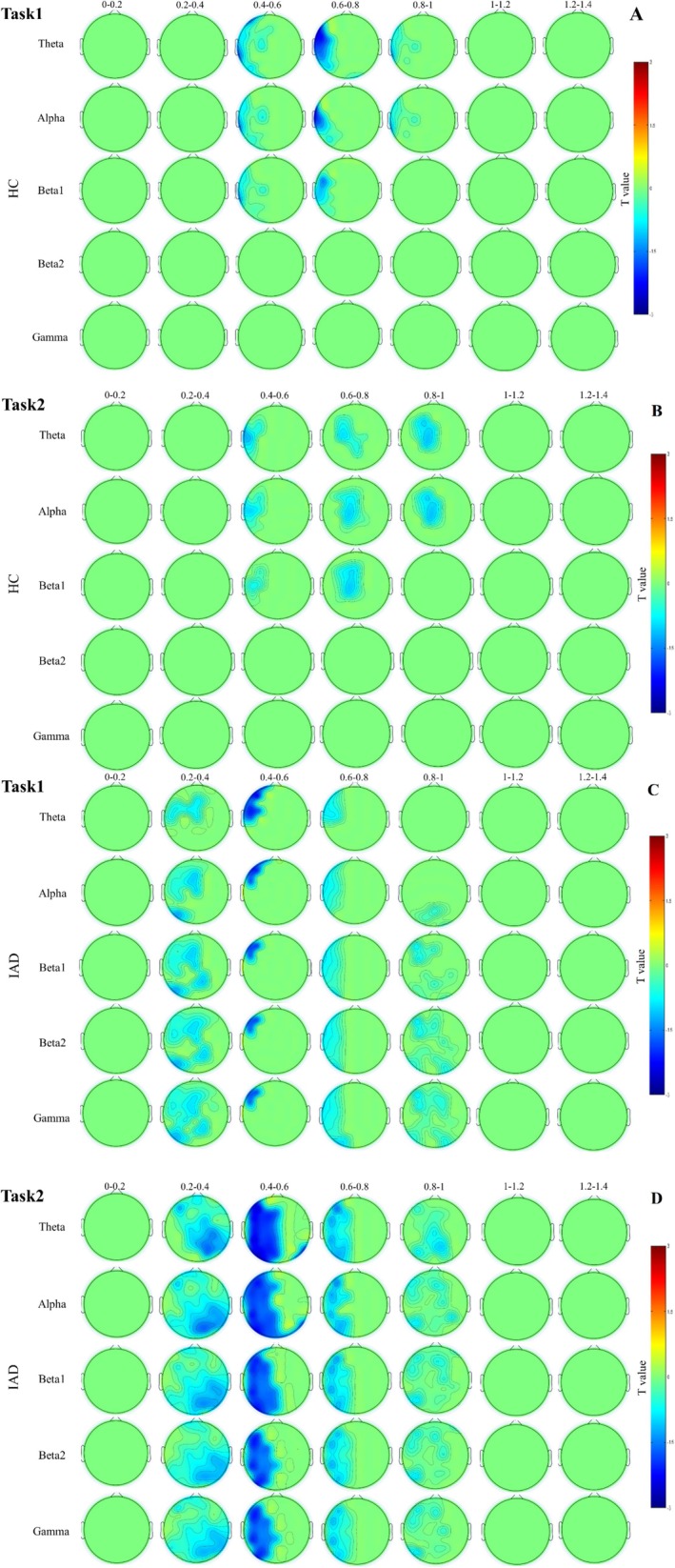
Scalp‐based ERSP results for both the HC group (A,B) and IAD group (C,D) across Task1 and Task2, obtained from basic impact analysis. Each row represents the mean value of a different frequency range, whereas each vertical division corresponds to the mean ERSP calculated at 200 ms intervals.

The scalp‐based ERSP analysis (Figure 10) examined Stroop effects in both groups across Task1 and Task2. In Task1, the IAD group showed ERSP Stroop effects primarily in the left hemisphere, spanning various frequency bands. Significant clusters emerged in the left fronto‐central and frontal regions between 200 and 800 ms, with additional effects in the central and fronto‐central regions from 200 to 400 ms. Beta1, beta2 and gamma activity were also observed between 800 and 1000 ms (Figure 10C). In contrast, the HC group in Task1 exhibited notable ERSP Stroop effects in early time windows, predominantly in the left hemisphere. Theta, alpha and beta1 activity appeared in fronto‐central, central and parietal regions between 600 and 800 ms, with a left‐lateralized cluster spanning 400–600 ms. Theta and alpha activity persisted in these regions from 800 to 1000 ms (Figure 10A).

In Task2, the IAD group displayed a strong ERSP Stroop effect, with a major cluster spanning theta, alpha, beta, and gamma frequencies in the left frontal region between 200 and 1000 ms (Figure 10D). The HC group exhibited widespread ERSP Stroop effects across frontal, central and parietal regions, with slight left lateralization. Significant theta, alpha and beta1 clusters appeared in fronto‐central and centro‐parietal regions between 400 and 600 ms, followed by another left‐lateralized cluster between 600 and 800 ms. Theta and alpha activity persisted in these regions from 800 to 1000 ms (Figure 10B).

## 4 Discussion

This study was designed to contrast conflict‐related ERPs and neural indicators associated with conflict‐monitoring processes in individuals with IAD and the HC group. In addition, it compared the spectral correlates of interference control in individuals with IAD with those of the HC group to identify potential differences. The colour‐word Stroop task and a modified version of the Stroop task were utilized during EEG recording, and time–frequency analysis was performed to estimate the neural activity underlying the time–frequency dynamics.

The findings indicated that IAD participants displayed a reduced conflict monitoring effect compared to HC participants, as demonstrated by the early and late MFN indices. Furthermore, individuals with IAD exhibited impaired conflict adaptation, which worsened as task difficulty increased, whereas the HC group maintained stable conflict adaptation. Theta‐band indices suggested that participants with IAD had reduced abilities to detect and respond to conflict compared to the HC group. Finally, gamma and beta2 frequency damage interaction patterns in the fronto‐central‐temporal areas further confirmed these impairments.

Behaviourally, all participants had longer RTs and higher ISD and made more errors on incongruent trials relative to congruent trials, which is consistent with Stroop‐related RT and error rate interference. Indeed, IAD individuals elucidated prolonged RTs compared to the HC group, with no significant differences in overall error rate or ISD. Our results are consistent with previous reports using computerized versions of the Stroop task in significant mean RT differences [13]. Moreover, the results in our modified version of the Stroop task showed generally higher ISD consistent with a previous study, along with delayed response times and especially significant differences between the two tasks in IAD individuals. Notably, the RT difference between groups was not uniformly present across all conditions but became significant as cognitive demands increased (i.e., incongruent trials and Task 2). This pattern suggests that the longer RTs in IAD individuals are not solely indicative of a general slowness but instead reflect greater difficulty in adapting to increased interference, supporting the presence of a conflict‐monitoring deficit. The findings suggest that IA may be linked to a deficit in inhibitory control, aligning with prior studies on substance use disorder and behavioural addiction [11, 44, 45].

A key observation in this study is the significant main effect of group on RTs, with IAD participants exhibiting generally slower responses than the HC group across conditions. Post hoc analyses revealed that the difference in RTs was most pronounced in incongruent trials and in Task2, suggesting that cognitive demands exacerbate performance deficits in IAD individuals. This pattern aligns with prior findings that individuals with executive function impairments exhibit greater deficits as task complexity increases [8].

Dong et al. [13] reported that impaired executive control in IAD individuals is reflected in lower MFN deflection during incongruent trials. Similarly, Buzzell et al. [20] found reduced N2 amplitude, a cognitive control marker, in smokers compared to non‐smokers. The ACC plays a key role in response inhibition, conflict detection and response monitoring [21]. Additionally, Claus et al. [46] linked conflict‐monitoring neural activity to alcohol use disorder severity, suggesting that inhibition and conflict processing may contribute to addiction vulnerability in youth. Whereas Dong et al. [13] found a positive association between ACC bold signal and RT in the Stroop task, our ERP results linked ACC activation to increased error commission. Incongruent trials elicited greater negative early and late MFN amplitudes than congruent trials, with the lower MFN amplitudes in IAD individuals indicating deficits in executive control and response inhibition. This study employed EEG to administer both the standard and modified Stroop tasks. Results showed a performance decline, marked by slower RTs and increased ISD, as task difficulty increased. IAD individuals exhibited weaker stimulus conflict detection than the HC group, as reflected by lower early MFN amplitudes in both tasks. However, within‐group comparisons revealed stable conflict detection in IAD individuals across tasks, whereas the HC group showed task‐dependent variation. This suggests that increased task difficulty impairs stimulus conflict detection in HC participants but not in IAD individuals. The early MFN, generated by the ACC in response to stimulus conflict [47], reflects conflict detection processes, with neuroimaging studies highlighting ACC and insula involvement in Stroop task performance [48].

The lower negative early and late MFN amplitudes in trials with longer RTs suggest reduced ACC activation during conflict processing in the IAD group, aligning with our behavioural finding that IAD individuals had significantly longer mean RTs than the HC group, despite no significant difference in error rates. The longer RTs may indicate slower conflict processing due to increased distractibility or less efficient cognitive resource allocation; however, maintained accuracy suggests that IAD individuals compensate with greater effort or delay. Although Su et al. [18] also reported impaired executive control in IAD individuals, indicated by a negative correlation between IAT score and MFN amplitude, they observed increased error rates in the IAD group, unlike our findings. This discrepancy may imply that, in our sample, IAD participants maintained accuracy despite slower responses, possibly by exerting more cognitive effort. Thus, the combination of longer RTs and lower MFN amplitudes points to attentional deficits in IAD individuals, reflecting reduced allocation of cognitive resources during conflict detection. Key executive functions include the ability to select a contextually appropriate response among numerous competing ones and the ability to suppress contextually inappropriate responses. These processes can be assessed through specific frontal and parietal ERP waves recorded during the processing of conflict response tendencies modified by the experimental task. ERP parameters related to response conflict detection and processing can be derived from frontal and fronto‐central ERP components such as late MFN. IAD participants demonstrated a lower degree of response conflict detection, as evaluated by late MFN mean amplitude, than HC participants in both tasks. However, response conflict detection significantly varied between the two tasks in IAD participants, but not in the HC group due to the insignificant task effect of late MFN in this group. The lower negative mean amplitude of the late MFN component in IAD participants than the HC group on Task1 indicates a decline in conflict response detection in IAD participants. However, this ability diminished significantly in IAD participants, but it remained unchanged in HC participants as task difficulty increased.

The late MFN is hypothesized to reflect selective attention to task‐relevant components by suppressing irrelevant information. Therefore, we expected impaired selective attention in IAD participants compared to HC participants, as task interference increased. Both early and late MFN components are generated in the ACC, and their amplitudes are enhanced with higher incongruity or interference, reflecting ACC activity during conflict monitoring [49]. Previous studies have shown that the late MFN corresponds to the period when inhibitory processes resolve Stroop interference, and impairments in the PFC in substance use disorders lead to difficulties in resolving response conflicts [50]. Our results showed that both early and late MFN amplitudes were consistently lower in IAD participants compared to HC group, aligning with Folstein and Van Petten [51], who associated reduced MFN amplitudes with impaired cognitive control. Importantly, as task difficulty increased, HC participants exhibited increased MFN amplitudes, indicating enhanced conflict detection and resolution, whereas IAD participants did not show this modulation. This suggests that IAD individuals have a diminished capacity to adapt their neural responses to increasing cognitive demands, reflecting impairments in selective attention and inhibitory control. These findings fit within the broader literature on neurocognitive deficits associated with addiction, highlighting the role of ACC and PFC dysfunction in impaired executive function.

The conflict SP, which is sustained positively over the parietal and centro‐parietal regions, is key to conflict resolution. The conflict SP is more positive on incongruent trials than congruent trials, leading to increased recruitment of cognitive control resources and compensation processes to prevent error commissions. This study found a significant difference in SP amplitude between IAD participants and HC participants in both tasks, indicating impaired conflict resolution in IAD participants. However, the ability to resolve conflict decreased with increasing task difficulty in IAD participants.

Proactive control strategies are crucial in determining how participants handle conflict. In situations where inhibitory control is needed, increased activity has been observed in the striatum, supplementary motor areas and midbrain [49]. According to the dual mechanisms of the cognitive control framework, interference processing can be interpreted by proactive and reactive modes of processing. SP was localized to the centro‐parietal and parietal brain regions as an indicator of proactive control. However, the present results suggest that proactive control (SP) was diminished in IAD participants.

We observed pronounced group differences in SP and early MFN components between IAD and HC participants. Specifically, both SP and early MFN amplitudes were significantly lower in IAD participants compared to HC participants, leading us to conclude that both proactive (SP) and reactive control modes (early MFN) are diminished in the IAD group. This suggests that making an extra effort to prepare in advance for the task at hand might enhance target‐related processing. In contrast, a lack of advance planning could cause task processing to take longer than intended [52].

Based on electrophysiological findings, we observed significant ERSP Stroop effects in the left frontal region of IAD individuals across theta, alpha, beta and gamma frequencies. This led to a more significant decrease in power on the left frontal regions in IAD participants when exposed to an incongruent situation compared to HC participants. This decrease was more pronounced across a wider range of channels and occurred at a higher frequency over a longer period [53]. As a result, we discovered that the ERSP Stroop effects were influenced by the group, with IAD participants showing earlier spectral correlates of interference control processes that were more extensive, less specific and more widespread than those observed in HC participants.

The Stroop effect, as observed in the HC group, was mainly noticeable in slow‐wave bands, indicating cognitive interference. This effect was seen in the left frontal region of the brain and gradually moved to the left frontal electrodes as the response time window closed. On the other hand, individuals with IAD recruited all frequency bands and exhibited low EEG power in the early phase, suggesting potential deficits in inhibiting the brain's conflict detection system [54]. As the task difficulty increased in Task2, the decrease in EEG power in the left hemisphere of IAD participants became more pronounced, indicating greater difficulty in suppressing brain activation for stimulus conflict under challenging conditions. This power reduction was distributed across the left hemisphere and affected a broader range of cortical areas.

Regarding the implications of spectral dynamics on cognitive tasks, theta band activity has been demonstrated to play a significant role in facilitating the organization of brain activity in general [55]. A common indicator of interference control and conflict resolution is oscillations in the theta band, which indicate a need for cognitive control. According to Qi et al. [56], overall theta band activity during incongruent trials is closely linked to conflict detection and response selection. Individuals with IAD exhibited theta band recruitment at an earlier time window and with reduced magnitude compared to the HC group. This suggests that conflict detection abilities were diminished earlier in IAD individuals following stimulus presentation as compared to HCs.

Compared to the HC group, individuals with IAD had an overrepresentation of beta2 and gamma frequencies, especially in Task2. This finding was significant compared to HC participants, as revealed by an extensive ERSP analysis. The activation pattern encompassed widespread bilateral regions, including the dorsal frontal and parietal cortices, without evidence of hemispheric asymmetry. This suggests that high‐frequency bands were involved in conflict detection and resolution in IAD participants in the early and late time windows. Consequently, IAD participants may require additional cognitive resources to manage conflict detection and resolution effectively. As task difficulty and interference levels increased in Task2, IAD participants exhibited additional activation in left posterior regions, which were not observed in HC participants. This suggests that the overrepresentation of beta2 and gamma bands in IAD individuals may reflect compensatory mechanisms to support cognitive processing under conditions of heightened task demands.

Our study aligns with previous research indicating that theta band activity changes are linked to conflict resolution processes. We found that in both congruent and incongruent situations, IAD individuals showed early suppression of theta band activity, hinting at their struggle with conflict stimuli detection. Furthermore, IAD participants displayed significant ERSP effects compared to HC participants at beta2 and gamma frequencies under incongruent conditions. These effects, absent in congruent conditions, suggest alterations in high‐frequency bands related to conflict detection and resolution.

Future research should investigate whether emotionally salient or reward‐related cues exacerbate inhibitory control deficits in IAD, as demonstrated in substance use disorders [57]. Moreover, variability in individual patterns of Internet use may contribute to differences in cognitive control, suggesting the need for studies that examine distinct IAD subtypes in relation to executive function. Additionally, whereas EEG provides high temporal resolution, integrating multimodal neuroimaging approaches, such as fMRI, could further elucidate the neural mechanisms underlying inhibitory control and conflict adaptation in IAD. These future directions will allow for a more refined characterization of cognitive control mechanisms in IAD and may inform targeted interventions aimed at improving executive function in individuals with problematic internet use.

This study is among the first to use conflict‐related ERPs and random permutation tests to investigate the neural indices underpinning conflict‐monitoring processes and oscillatory dynamics in conflict processing for IAD individuals. Findings suggest that individual with IAD have diminished capabilities in detecting conflict stimuli and responses, as indicated by early and late MFN indices. Our research also revealed that individuals with IAD exhibited alterations in neural oscillations due to cognitive impairments associated with theta, beta2 and gamma frequencies. This suggests that IAD may affect various cognitive processes involved in conflict monitoring and processing, contributing to the behavioural deficits observed in this study. By employing a modified version of the task (Task2) specifically for the IAD individuals, we were able to more effectively differentiate the neural indices underlying conflict‐monitoring processes and oscillatory dynamics in conflict processing between the IAD group and the HC groups. Task2, with greater cognitive demands and higher interference, provided a more sensitive measure of impairments in conflict processing among IAD participants, offering insights for future interventions and treatments aimed at addressing cognitive deficits associated with IAD.

## Author Contributions

**Farzad Rostami:** conceptualization, methodology, formal analysis, investigation, data curation, writing – original draft, writing – review and editing, visualization. **Ali Esteki:** conceptualization, supervision, project administration. **Sepideh Khoniveh:** investigation, data curation. **Rana Ghamari:** conceptualization. **Atiye Sarabi‐Jamab:** methodology, writing – review and editing, supervision, project administration.

## Conflicts of Interest

The authors declare no conflicts of interest.

## Supporting information


**Table S1** The average amplitude of early MFN across seven frontal electrodes (F1, F2, Fz, FC1, FC2, FCz and Cz) compared between the IAD and HC groups across congruent and incongruent trials in both Task1 and Task2.
**Table S2.** The average amplitude of late MFN across seven frontal Medline electrodes (F1, F2, Fz, FC1, FC2, FCz and Cz) compared between the IAD and HC groups across congruent and incongruent trials in both Task1 and Task2.
**Table S3.** The average amplitude of SP across centro‐parietal, and parietal electrodes (P3, P4, Pz, CP3, CP4 and CPz) compared between the IAD and HC groups across congruent and incongruent trials in both Task1 and Task2.

## Data Availability

The data that support the findings of this study are available from the corresponding author upon reasonable request.
