# Supplementary material for: Impaired Interference Control in Individuals With Internet Addiction: Evidence From Event‐Related Potentials and Brain Oscillations
Source: Addict Biol. 2025 Jul 24;30(7):e70062. doi: 10.1111/adb.70062 (PMC12290022; doi:10.1111/adb.70062)
Supplement: Supplementary file 1 — Table S1 The average amplitude of early MFN across seven frontal electrodes (F1, F2, Fz, FC1, FC2, FCz and Cz) compared between the IAD and HC groups across congruent and incongruent trials in both Task1 and Task2. Table S2. The average amplitude of late MFN across seven frontal Medline electrodes (F1, F2, Fz, FC1, FC2, FCz and Cz) compared between the IAD and HC groups across congruent and incongruent trials in both Task1 and Task2. Table S3. The average amplitude of SP across centro‐parietal, and parietal electrodes (P3, P4, Pz, CP3, CP4 and CPz) compared between the IAD and HC groups across congruent and incongruent trials in both Task1 and Task2. [file ADB-30-e70062-s001.docx]

**Supplementary Material**

## **Additional Methodological Details**

**Data Acquisition and Preprocessing** In addition to the procedures described in the main text, further details regarding EEG data acquisition and preprocessing are provided to enhance reproducibility. EEG signals were recorded using a 64-channel g.HIamp amplifier system (g.tec Medical Engineering GmbH, Austria), with electrodes placed according to the international 10–10 system to ensure comprehensive scalp coverage. The right mastoid electrode served as the online reference, and the ground electrode was positioned at Fpz. Electrode impedances were maintained below 10 kΩ throughout the recording session to ensure high-quality signal acquisition.

The raw EEG data were digitized at a sampling rate of 1,200 Hz. Preprocessing was conducted using EEGLAB v.2021.0 (1). A band-pass filter ranging from 0.5 to 40 Hz was applied to eliminate low-frequency drifts and high-frequency noise. Artifact rejection involved both automatic detection and visual inspection to identify and exclude segments contaminated by muscle activity, eye movements, or other artifacts. Noisy channels were identified based on their statistical properties and were interpolated using spherical spline interpolation to preserve the spatial structure of the data. Independent Component Analysis (ICA) was employed to separate and remove residual artifacts such as eye blinks and cardiac signals. ICA components representing artifacts were identified by their characteristic time courses and scalp topographies and were subsequently removed from the data.

Following artifact correction, the EEG data were downsampled to 256 Hz to reduce computational demands without significant loss of information. Epochs were extracted from the continuous data, spanning from 200 milliseconds before to 1,500 milliseconds after stimulus onset. Baseline correction was applied using the pre-stimulus interval (−200 to 0 milliseconds) to account for pre-existing voltage differences. Epochs exceeding ±100 μV in any channel were considered artifacts and excluded from further analysis. On average, approximately 122 trials per condition in Task 1 and 61 trials per condition in Task 2 were retained after preprocessing, ensuring sufficient data quality for reliable ERP and time-frequency analyses. The ERPs were generated using ERPLAB (2) by averaging the individual artifact-free trials across participants for each condition (congruent and incongruent) and task (Task 1 and Task 2). Grand averages were then computed for each group over the selected electrodes to facilitate group-level analyses.

**Time-Frequency Analysis**

To investigate oscillatory brain activity associated with cognitive control and interference processing, time-frequency analysis was conducted using Morlet wavelet transformations. The wavelet decomposition covered frequencies ranging from 4 to 40 Hz, with a resolution of 1 Hz. The number of cycles in the wavelets increased linearly from 2 at 4 Hz to 20 at 40 Hz, providing an optimal balance between time and frequency resolution across the spectrum. Baseline correction for the ERSPs was achieved by subtracting the mean power during the pre-stimulus interval from each time point in the post-stimulus period. The analysis focused on frequency bands relevant to cognitive control processes, including theta (4-7 Hz), alpha (8-12 Hz), beta (13-30 Hz), and gamma (31-40 Hz). These frequency bands were selected based on previous research (33, 44, 87). The beta band was then partitioned into two distinct portions, with frequencies ranging from 13 to 19 Hz (referred to as beta1) and 20 to 30 Hz (referred to as beta2) (3). The time-frequency power estimates for each subject, condition, block, location, and frequency band (theta, alpha, beta1, beta2, and gamma) were averaged by employing wavelet representations of individual trials. Post-stimulus power changes were measured as a change from baseline (from 200 to 0 ms after stimulus onset). In contrast to the event-related potential method, this particular methodology offers insights into the temporal dynamics of distinct frequency bands' engagement in certain cognitive activities (4). By employing this methodology for the examination of brain oscillations, it becomes feasible to discern cognitive processes linked to neural oscillations through the extraction of pertinent data concerning alterations in power across various frequencies and their temporal fluctuations (referred to as the extraction of Event-Related Spectral Perturbations, or ERSP).

**Statistical analysis**

To control for multiple comparisons in the EEG data, cluster-based random permutation tests were employed (5). This non-parametric approach increases statistical power by considering the spatial and temporal correlations in the data while effectively controlling the family-wise error rate. For the ERSP analysis, statistical comparisons were made between conditions and groups at each time-frequency point across all electrodes. Clusters of significant differences were identified by grouping adjacent time points, frequencies, and electrodes that exceeded a predefined threshold (p < 0.05). The cluster-level statistics were calculated by summing the t-values within each cluster. A null distribution was generated by randomly permuting the condition or group labels across participants 1,000 times. This permutation procedure was conducted under the null hypothesis of no difference between conditions or groups. The cluster-level statistics from each permutation were computed to form the null distribution. Clusters in the original data were considered significant if their statistics exceeded the critical values derived from the null distribution, corresponding to a two-tailed alpha level of 0.05. This statistical method was first applied to assess the ERSP Stroop effect (incongruent vs. congruent conditions) within each group. Subsequently, between-group comparisons were conducted to examine differences in neural oscillatory responses associated with cognitive interference between the IAD group and the HC group. By employing this approach, the analysis accounted for the multiple comparisons problem inherent in EEG data without overemphasizing specific data points or introducing biases.

**Results**

**RT**

For the analysis of mean RTs, trials with incorrect responses and those exceeding mean_RTs±2SD were excluded. According to post-hoc ANOVA subgroup analysis, the effect of the group in congruent trials was found to be statistically significant (*p = 0.021,* $\eta_{p}^{^{2}}$ *= 0.074*) , with the HC group showing the shortest mean RT and the IAD group the longest mean RT. Moreover, in the incongruent trials, a significant main impact of group was found (*p < 0.001,* $\eta_{p}^{^{2}}$ *= 0.157*), primarily due to the longer mean RTs observed in individuals with IAD compared to HC group. Furthermore, a signiﬁcant difference was found in Task1 between the IAD and HC groups (*p = 0.012,* $\eta_{p}^{^{2}}$ *= 0.053*) and also between congruent and incongruent trails (*p < 0.001,* $\eta_{p}^{^{2}}$ *= 0.279*). Similarly, a significant difference was found in Task2 between IAD and HC groups (*F (1, 57) = 11.96, p < 0.001,* $\eta_{p}^{^{2}}$ *= 0.142*) and also between congruent and incongruent trails (*F (1, 57) = 24.19, p < 0.001,* $\eta_{p}^{^{2}}$ *= 0.180*). Within the IAD group, RTs in incongruent trials were significantly longer than in congruent trials (*p < 0.001,* $\eta_{p}^{^{2}}$ *= 0.313*) and there was a marginally significant difference between Task1 and Task2 (*p = 0.0788,* $\eta_{p}^{^{2}}$ *= 0.031*). Within the HC group, RTs in incongruent trials were also significantly longer than in congruent trials (p < 0.001, $\eta_{p}^{^{2}}$ = 0.195), but no significant difference was observed between Task1 and Task2. (*p = 0.761*).

**ISD**

To analyze ISD, outliers were included to assess the full range of RT variation. In the post-hoc analysis, separate ANOVAs were conducted to further examine the main effects found in the initial analysis. The main effect of task was significant in both congruent trials (*p = 0.002,* $\eta_{p}^{^{2}}$ *= 0.123*), resulting in higher speed variability of Task2 than Task1 in all trials. However, there was no significant group effect in congruent (*p = 0.307*) and incongruent trials (*p = 0.654*). Additionally, a significant congruity effect was found in task1 (*p < 0.001,* $\eta_{p}^{^{2}}$ *= 0.356*) and task2 (*p < 0.001,* $\eta_{p}^{^{2}}$ *= 0.322*). The task effect was only significant in the IAD group (*p < 0.001,* $\eta_{p}^{^{2}}$ *= 0.153*), reflecting the greater variation in response speed in this group.

**ERP Data**

In this study, the distribution of grand-averaged ERP activity was analyzed. The early MFN, late MFN, and SP components were calculated using a pre-stimulus baseline window from −200 ms to 0 ms and measured as mean amplitudes within specific timeframes. For the early and late MFN components, mean amplitudes were calculated over the frontal, fronto-central, and central electrodes: F1, F2, Fz, FC1, FC2, FCz, and Cz. The SP component was calculated over the centro-parietal and parietal electrodes: CP3, CPz, CP4, P3, Pz, and P4.

**Early MFN (Table S1)**

Regarding the early MFN component, its calculation involved the frontal, fronto-central, and central regions. A significant difference was observed between the IAD and HC groups in both Task1 (p < 0.001, $\eta_{p}^{^{2}}$ = 0.093) and Task2 (p < 0.001, $\eta_{p}^{^{2}}$ = 0.034). Specifically, within the IAD group, a significant difference was found between congruent and incongruent trials (p < 0.01, $\eta_{p}^{^{2}}$ = 0.014), whereas no such difference was observed in the HC group (p = 0.526). Additionally, no significant congruity effect was found in Task1 (p = 0.302), while a significant congruity effect was observed in Task2 (p < 0.01, $\eta_{p}^{^{2}}$ = 0.018). Notably, within the IAD group, no significant difference was observed between Task1 and Task2 (p = 0.526), whereas a significant difference was found in the HC group (p = 0.01, $\eta_{p}^{^{2}}$ = 0.014).

**Table S1. The average amplitude of Early MFN across seven frontal electrodes (F1, F2, Fz, FC1, FC2, FCz, Cz) compared between the IAD and HC groups) across congruent and incongruent trials in both Task1 and Task2**

|  | Early MFN | | | | | | | |
| --- | --- | --- | --- | --- | --- | --- | --- | --- |
|  | Task1 | | | | Task2 | | | |
|  | Congruent | | Incongruent | | Congruent | | Incongruent | |
|  | HC | IAD | HC | IAD | HC | IAD | HC | IAD |
| F1 | -1.52(1.03) | -0.41(3.03) | -2.15(1.36) | -0.50(3.55) | -2.10(1.83) | -0.13(2.49) | -2.43(2.04) | -0.21(2.72) |
| F2 | -1.48(1.21) | -0.21(2.90) | -1.98(1.38) | -0.25(3.09) | -2.35(1.78) | -0.28(2.44) | -2.80(1.95) | -0.50(2.63) |
| Fz | -1.39(1.19) | -0.34(2.90) | -2.02(1.52) | -0.44(3.43) | -2.03(2.54) | -0.17(1.80) | -2.25(2.01) | -0.07(2.69) |
| FC1 | -1.70(1.72) | -0.12(3.49) | -2.54(2.14) | -0.06(4.28) | -2.76(2.29) | -0.43(3.04) | -3.25(2.81) | -0.61(3.04) |
| FC2 | -2.40(1.90) | -0.76(3.14) | -3.12(1.82) | -0.82(3.64) | -3.41(2.31) | -0.83(2.79) | -3.83(2.69) | -1.07(2.79) |
| FCz | -1.64(2.18) | -0.20(3.29 | -2.44(2.38) | -0.32(4.13) | -2.77(2.47) | -0.33(2.99) | -3.22(2.79) | -0.64(2.99) |
| Cz | -3.87(2.72) | -1.91(3.77) | -4.56(2.48) | -1.83(4.17) | -4.53(3.07) | -1.94(3.52) | -4.93(3.70) | -2.03(3.21) |

**Late MFN (Table S2)**

For the late MFN component, the negative mean amplitude was compared between congruent and incongruent trials in both tasks. A significantly larger negative mean amplitudes were found for incongruent trials compared to congruent trials in both Task1 (*p = 0.007,* $\eta_{p}^{^{2}}$ *= 0.012*) and Task2 (*p = 0.017,* $\eta_{p}^{^{2}}$ *= 0.031*). Furthermore, a significant difference was observed between the IAD and HC groups in both Task1 (*p = 0.001,* $\eta_{p}^{^{2}}$ *= 0.027*) and Task2 (*p < 0.001,* $\eta_{p}^{^{2}}$ *= 0.029*), with the IAD group exhibiting lower negative mean amplitudes of the late MFN compared to the HC group. A significant congruity effect was found within both the IAD (*p = 0.016,* $\eta_{p}^{^{2}}$ *= 0.042*) and HC (*p = 0.019,* $\eta_{p}^{^{2}}$ *= 0.013*) groups. Additionally, within the IAD group, a significant difference was found between Task1 and Task2 (*p = 0.04,* $\eta_{p}^{^{2}}$ *= 0.008*), whereas within the HC group, no significant difference was revealed between the two tasks (*p = 0.187*).

**Table S2. The average amplitude of late MFN across seven frontal Medline electrodes (F1, F2, Fz, FC1, FC2, FCz, Cz) compared between the IAD and HC groups) across congruent and incongruent trials in both Task1 and Task2**

|  | Late MFN | | | | | | | |
| --- | --- | --- | --- | --- | --- | --- | --- | --- |
|  | Task1 | | | | Task2 | | | |
|  | Congruent | | Incongruent | | Congruent | | Incongruent | |
|  | HC | IAD | HC | IAD | HC | IAD | HC | IAD |
| F1 | -1.76(1.52) | -0.37(3.05) | -2.35(1.78) | -1.19(3.32) | -1.49(1.58) | -0.50(3.36) | -2.47(1.82) | -1.39(3.44) |
| F2 | -1.93(1.28) | -0.29(2.69) | -2.49(1.64) | -1.07(3.06) | -1.94(1.46) | -0.67(3.32) | -2.73(1.67) | -1.43(3.42) |
| Fz | -1.69(2.63) | -0.17(2.93) | -2.22(2.03) | -0.95(3.34) | -1.36(1.66) | -0.26(3.48) | -2.30(1.93) | -1.13(3.50) |
| FC1 | -2.82(2.51) | -0.41(2.80) | -3.69(2.75) | -1.36(4.12) | -2.48(2.23) | -0.92(4.25) | -3.63(2.68) | -1.98(4.06) |
| FC2 | -2.61(2.13) | -0.14(2.39) | -3.45(2.39) | -1.07(2.55) | -3.15(2.26) | -1.17(4.04) | -4.10(2.55) | -2.17(3.87) |
| FCz | -2.48(2.79) | -0.12(3.75) | -3.31(3.05) | -1.23(4.06) | -2.43(2.46) | -0.79(4.29) | -3.51(2.79) | -1.92(4.08) |
| Cz | -4.03(3.22) | -1.12(4.17) | -5.01(3.34) | -2.02(4.05) | -4.10(3.28) | -2.11(4.90) | -5.31(3.73) | -3.23(4.33) |

**SP (Table S3)**

For the SP component, which was calculated over the centro-parietal and parietal areas, post-hoc analysis revealed significant differences between the IAD and HC groups in both congruent (*p < 0.001,* $\eta_{p}^{^{2}}$ *= 0.048*) and incongruent (*p < 0.001,* $\eta_{p}^{^{2}}$ *= 0.052*) trials. Within the IAD group, a significant difference was observed between congruent and incongruent trials (*p = 0.042,* $\eta_{p}^{^{2}}$ *= 0.012*), while no significant task effect was found in either trial type (congruent: (p = 0.47), incongruent: (*p = 0.151*)). Furthermore, a marginally significant main effect of task was found in the IAD group (*p = 0.068,* $\eta_{p}^{^{2}}$ *= 0.007*), along with a significant main effect of congruity (*p = 0.012,* $\eta_{p}^{^{2}}$ *= 0.017*). No other main effects were observed in either the IAD or HC groups. A significant group effect was found in both Task1 (*p = 0.004,* $\eta_{p}^{^{2}}$ *= 0.026*) and Task2 (*p < 0.001,* $\eta_{p}^{^{2}}$ *= 0.075*), along with a marginally significant congruity effect in Task1 (*p = 0.068,* $\eta_{p}^{^{2}}$ *= 0.006*) and a significant congruity effect in Task2 (*p = 0.042,* $\eta_{p}^{^{2}}$ *= 0.012*).

**Table S3. The average amplitude of SP across centro-parietal, and parietal electrodes (P3, P4, Pz, CP3, CP4, CPz) compared between the IAD and HC groups across congruent and incongruent trials in both Task1 and Task2**

|  | SP | | | | | | | |
| --- | --- | --- | --- | --- | --- | --- | --- | --- |
|  | Task1 | | | | Task2 | | | |
|  | Congruent | | Incongruent | | Congruent | | Incongruent | |
|  | HC | IAD | HC | IAD | HC | IAD | HC | IAD |
| P3 | 2.67(1.07) | 0.14(2.10) | 3.35(1.71) | 0.69(2.40) | 2.25(1.31) | 1.21(2.79) | 3.49(1.36) | 1.93(2.99) |
| P4 | 2.42(1.37) | 0.68(1.45) | 3.01(1.56) | 1.15(1.54) | 2.21(2.06) | 0.88(2.10) | 3.01(2.15) | 1.72(1.82) |
| Pz | 2.95(1.47) | 0.98(1.84) | 3.63(2.18) | 1.62(2.07) | 2.52(1.90) | 1.74(2.71) | 3.85(2.10) | 2.27(2.99) |
| CP3 | 4.12(1.81) | 2.63(2.33) | 4.51(2.06) | 3.10 (2.67) | 3.95(1.50) | 3.17(3.51) | 5.13(2.22) | 3.67(3.73) |
| CP4 | 4.25(1.41) | 2.62(1.78) | 4.61(1.77) | 3.02 (2.04) | 4.23(1.73) | 3.15(2.79) | 4.90(1.87) | 3.72(3.23) |
| CPz | 4.55(1.97) | 3.11(2.53) | 5.09(2.37) | 3.74(3.11) | 4.50(1.73) | 3.84(3.81) | 5.63(2.53) | 4.26(3.37) |

**Scalp-based time-frequency**

For a more comprehensive understanding of the ERSP analysis results, refer to Figure 9. Each row represents the average for a frequency band, while each column represents the average ERSP computed every 200 ms.

The IAD group showed the first major cluster with ERSP Stroop effects in the theta, alpha, beta, and gamma frequencies in Task1. This cluster was recorded across multiple electrodes in the left frontal area and lasted between 200 and 1000 ms. These samples also included earlier time windows. Compared to the HC group, IAD participants demonstrated a weaker ERSP Stroop effect , as evidenced by reduced event-related power changes. A stronger event-related decrease in power was observed by IAD participants during the incongruent condition compared to HC participants, primarily due to the weaker event-related decrease on the left frontal electrodes. Additionally, another cluster of beta, alpha, and gamma bands was detected across the left posterior channels in an earlier time window spanning 200 to 400 ms and 600 to 1000 ms (Figure 9-C). Notably, for all frequency bands except theta, the power increase during the congruent condition was greater in the IAD participants compared to the HC group, implying distinct effects between the two groups.

During Task2, in the IAD group, a significant cluster of ERSP Stroop effects emerged across over the left hemisphere in a time window of approximately 400 to 800 ms. Additionally, a similar pattern was observed between 200 to 400 ms across most scalp channels, excluding the left temporal and lateral frontal channels. Within a time window of 800 to 1000 ms, a left- lateralized effect was detected across the central and frontal regions. A second cluster, encompassing theta and alpha frequencies, was found over bilateral posterior channels, along with beta1, beta2, and gamma bands over the left posterior channels during time windows from 800 to 1000 ms (Figure 9-D).

**References**

1. Delorme A, Makeig S. EEGLAB: an open source toolbox for analysis of single-trial EEG dynamics including independent component analysis. Journal of neuroscience methods. 2004;134(1):9-21.

2. Lopez-Calderon J, Luck SJ. ERPLAB: an open-source toolbox for the analysis of event-related potentials. Frontiers in human neuroscience. 2014;8:213.

3. Nigbur R, Ivanova G, Stürmer B. Theta power as a marker for cognitive interference. Clinical Neurophysiology. 2011;122(11):2185-94.

4. Engel AK, Fries P. Beta-band oscillations—signalling the status quo? Current opinion in neurobiology. 2010;20(2):156-65.

5. Maris E, Oostenveld R. Nonparametric statistical testing of EEG-and MEG-data. Journal of neuroscience methods. 2007;164(1):177-90.
